# Hypochondriac concerns and correlates of personality styles and affective states in bipolar I and II disorders

**DOI:** 10.1186/s12888-018-1988-0

**Published:** 2018-12-22

**Authors:** Bing Pan, Qing Zhang, Huitzong Tsai, Bingren Zhang, Wei Wang

**Affiliations:** 1 Department of Psychiatry, Second Affiliated Hospital, Zhejiang University College of Medicine, Hangzhou, China; 2 Department of Clinical Psychology and Psychiatry/ School of Public Health, Zhejiang University College of Medicine, Zhejiang, 310058 Hangzhou China

**Keywords:** Affective states, Bipolar I and II disorders, Hypochondriasis, Illness attitude scales, Personality disorder functioning styles

## Abstract

**Background:**

Hypochondriac concerns are associated with the treatment-difficulty of bipolar disorder, which might be due to the personality styles and affective states.

**Methods:**

We invited outpatients with bipolar I disorder (BD I, *n* = 87), bipolar II disorder (BD II, *n* = 92) and healthy volunteers (*n* = 129) to undergo the Illness Attitude Scales and Parker Personality Measure tests, and measurements of concurrent affective states**.**

**Results:**

Compared to healthy volunteers, BD I and BD II patients scored significantly higher on mania, hypomania and depression. BD I and BD II patients also scored significantly higher on Symptom Effect and Treatment Seeking, and BD II patients scored higher on Patho-thanatophobia and Hypochondriacal Belief. BD II in addition scored higher on Patho-thanatophobia than BD I did. In controls, the Dependent style predicted Patho-thanatophobia and Symptom Effect, Schizoid with Hypochondriacal Belief; in BD I, Narcissistic (−) with Hypochondriacal Belief, Histrionic with Patho-thanatophobia and Hypochondriacal Belief, depression with Hypochondriacal Belief, and hypomania with Symptom Effect and Hypochondriacal Belief; in BD II, depression with Symptom Effect and Hypochondriacal Belief, mania with Symptom Effect**.**

**Conclusions:**

Bipolar disorder, especially BD II, is associated with greater hypochondriac concerns, which relates to personality disorder functioning styles and concurrent affective states.

## Introduction

Bipolar disorder has two major types, i.e., bipolar I (BD I) and II (BD II) disorders. Both are devastating and are characterized by recurrent episodes of mania/ hypomania and depression [1], with frequent relapse, lingering residual symptoms, impaired cognition, and diminished wellbeing [2]. In particular, the BD II exhibits more serious symptoms [3], more chronicity [4], and more comorbidity with physical illness, anxiety, and personality disorders than BD I [5]. The poor prognoses of BD I and BD II are related to the poor adherences to treatment, the anxious attitudes and beliefs towards health [6].

The hypochondriasis is a preoccupation of having or acquiring a serious illness, which is frequently defined as a chronic condition distinctive from affective disorders [7] or is referred as health anxiety disorder [8], illness anxiety disorder, or somatic symptom disorder [9]. Specifically, there are several aspects of hypochondriasis as measured with the Illness Attitude Scales (IAS), which is considered one of the most suitable instruments for hypochondriasis screening and to discriminate hypochondriac patients from healthy volunteers [10, 11]. Recently, Luo et al. [12] have validated the IAS structures into four factors: Patho-thanatophobia reflecting the fears of serious illness or death, Symptom Effect describing the effects of symptoms on everyday life and work, Treatment Seeking reflecting the action of disease treatment and prevention, and Hypochondriacal Beliefs representing the doubting of being healthy despite medical reassurance.

However, the exact role of the hypochondriasis played in BD I and BD II etiopathology is unknown. The implicit theories might offer some explanation for the hypochondriasis in the disorder. According to the theories, the belief is connected with cognition and behavior in psychological processes, while the maladapted belief is related to the onset and maintenance of anxiety and depression in psychiatric patients in particular [13, 14]. In clinics, some patients with major depressive disorder also met the diagnosis of hypochondriasis [15]. Compared to patients without hypochondriasis, depressed patients who are comorbid with hypochondriasis display more somatic symptoms, higher suicidal attempts, and more treatment-seeking [16–18]. Increased manic episodes during a lifetime are associated with an elevated hypochondriasis score in BD I [19]. In bipolar spectrum patients, the perception of their overall physical health can predict mania/ hypomania and depression severity levels, and life satisfaction at concurrent and future visits [20]. Meanwhile, as an ego defense, hypochondriasis was reported to often signal an imminently affective change in bipolar disorder patients, especially when the switch out of mania was started [21]. Moreover, BD II displays more depressive symptoms than BD I [3]. However, whether different affective states affect the hypochondriasis differently in BD I and BD II remains uncertain.

Again according to the implicit theories, personality trait plays an important role in regulating decision-making and behavioral reactions of an individual [13], and it might also be related to the etiopathology of hypochondriasis. For instance, one study reported that 76.5% patients with hypochondriasis had one or more personality disorders, with common disorders being obsessive-compulsive and avoidant types [22]. Another study indicated that patients with hypochondriasis presented characteristics of paranoid, borderline, avoidant, and dependent personality disorders [23]. Interestingly, bipolar disorder patients display some unique personality traits or personality disorders. For example, the neuroticism and borderline personality disorder functioning style (trait) were reportedly higher in BD II than those in BD I [23, 24], and the impulsive sensation seeking trait was lower in BD II than in BD I [25]. The borderline style predicted depression and mania in BD I and BD II, antisocial predicted hypomania in BD I, and passive-aggressive predicted hypomania in BD II [24]. Nonetheless, it is unclear whether the involvements of antisocial, borderline, histrionic, narcissistic, or other disordered personality symptoms in hypochondriasis are different in BD I and BD II.

Therefore, it is reasonable to speculate that both affective states and personality disorder functioning styles affect hypochondriasis in both BD I and BD II. In the current study, we hypothesized that: (1) the bipolar disorder patients score higher than healthy volunteers do on the psychopathological variables, and BD II patients score higher than BD I patients do on most aspects of hypochondriasis; and (2) the associations between the hypochondriac concerns and depression and mania, or the antisocial, borderline, histrionic, and narcissistic personality disorder styles are different in the two types of bipolar disorder. Thus, we used the Chinese version of IAS [12] to measure different aspects of hypochondriasis, besides applying the measurements for personality disorder functioning styles and concurrent affective states in our participants.

## Methods

### Participants

We enrolled 129 healthy participants (58 men; mean age, 24.55 ± 3.48 years; age range, 16 ~ 49 years), who were physically healthy and had no history of psychiatric or neurological abnormalities. We also invited 241 outpatients with bipolar disorder, but later 27 patients comorbid with personality disorder(s), and 35 with the complex somatic symptom disorder/ illness anxiety disorder, were excluded. Finally, 87 with BD I (41 men; mean age, 25.15 ± 7.95 years; range, 16 ~ 45 years) and 92 with BD II (33 men; mean age, 25.27 ± 6.83 years; range, 16 ~ 49 years) were enrolled, who were diagnosed according to the Diagnostic and Statistical Manual of Mental Disorders, Version 5 (DSM-5) [9], by two experienced psychiatrists (PB and WW). In BD I, 25 patients were in depression state, 21 in mania state, one in mixed state, and 40 in remission; In BD II, 30 were in depression state, 24 in hypomania state, and 48 in remission.

A recent computed tomography or magnetic resonance imaging scan was available to ensure that all patients were free from any organic brain lesions. All patients were also confirmed to have no other confounding factors including schizophrenia, schizoaffective disorder, or prior history of head injury, alcohol, or tobacco abuse, psychoactive substance abuse, central nervous system inflammation, or neurocognitive or other disorders influencing their decisional capacity (understanding, appreciation, reasoning, and expression of action choice) through the semistructured clinical interview. Moreover, all participants completed their 9 years of education. No significant differences were found among the three groups regarding gender (χ^2^ = 2.71, *p* = .26) or age (F [2, 308] = .46, *p* = .64, mean square effect (MSE) = 16.76) distributions. Participants had to be free from alcohol and other substances for at least 72 h prior to testing. The study protocol was approved by the Medical Ethics Committee of School of Public Health, Zhejiang University. All participants provided their written informed consent, and guardians signed the written informed consent for the adolescents or bipolar disorder patients.

### Measures

#### Illness Attitude Scale (IAS)

The IAS is a scale measuring medically unexplained physical symptoms relating to hypochondriasis [10, 26]. Its Chinese version is comprised of 27 items rated on a five-point scale (0 - no, 1 - rarely, 2 - sometimes, 3 - often, 4 - most of the time) that measuring four factors of hypochondriasis. Patho-thanatophobia (internal alpha, .82), Symptom Effect (.82), Treatment Seeking (.74), And factor 4, Hypochondriacal Beliefs (.68) [12].

#### Parker Personality Measure (PERM)

The PERM is a self-report questionnaire helping identify types of personality disorder. It consists of 92 items each with a five-point Likert scale (1 - very unlike me, 2 - moderately unlike me, 3 - somewhat unlike and like me, 4 - moderately like me and 5 - very like me) [27]. The questionnaire assesses 11 personality disorders: paranoid (internal alpha, .70), schizoid (.35), schizotypal (.62), antisocial (.68), borderline (.78), histrionic (.55), narcissistic (.70), avoidant (.75), dependent (.72), obsessive-compulsive (.50), and passive-aggressive (.68) types [28].

#### Mood Disorder Questionnaire (MDQ)

The MDQ consists of three parts comprising13 forced-choice (yes or no) questions [29]. It assesses the presence of symptoms and behaviors related to mania or hypomania, one question to determine whether two or more symptoms have been experienced at the same time, and one question to determine the extent to which symptoms have caused functional impairment on a scale ranging from “no problems” to “serious problems”. Its internal reliability was .79 in a Chinese sample [30].

#### Hypomania Checklist-32 (HCL-32)

The HCL-32 consists of 32 items [31]. Individuals were instructed to answer the forced-choice (yes or no) questions about emotions, thoughts, or behaviors, and to answer questions regarding the duration, the impact on family, social and work life, or people’s reactions. Its internal reliability was .88 in a Chinese sample [32].

#### Plutchik - van Praag Depression Inventory (PVP)

The PVP is a self-assessment instrument including 34 items for detecting depressive symptoms [33]. Each item has three scale points (0, 1, and 2) corresponding with increasing depressive tendencies. Participants have “possible depression” if they score between 20 and 25, or “depression” if they score above 25. The internal reliability of the inventory was .94 in a Chinese sample [34].

### Statistical analyses

Two-way ANOVA was applied to the mean IAS and PERM scale scores, and one-way ANOVA to the mean scores of PVP, MDQ, and HCL-32 in the three groups of participants. Later, the effect sizes (the eta squared values) of ANOVA were calculated. Whenever a significant main effect was found, post-hoc analysis by the Bonferroni test was employed to evaluate between-group differences. In addition, we applied the stepwise multiple linear regression analysis (backward method) to search for the relationships between the IAS, PERM, PVP, MDQ, and HCL-32 scales, taking PERM styles and affective states as potential predictors for IAS factors. A *p*-value < .05 (in ANOVA) or .01 (in prediction, to prevent false positives) was considered significant.

## Results

### Group differences of illness attitudes (hypothesis 1) and personality styles

The mean IAS scores were significantly different among the three groups (F [2, 304] = 13.84, *p* < .001, MSE = 730.75, ŋ^2^ = .08). Post-hoc analyses showed that BD I scored significantly higher than controls did on Symptom Effect and Treatment Seeking; BD II scored significantly higher than controls did on Patho-thanatophobia, Symptom Effect, Treatment Seeking and Hypochondriacal Belief. In addition, BD II scored significantly higher than BD I did on Patho-thanatophobia (Table 1). The mean PERM scale scores were significantly different among the three groups (F [2, 305] 1 = 44.98, *p* < .001, MSE =8039.75, ŋ^2^ = .23). Post-hoc analyses showed that BD I and BD II scored significantly higher than the controls did on most scales. BD II also scored significantly higher than controls did on Schizoid. BD II scored significantly higher on Borderline, Schizotypal and Dependent scales than BD I did (Table 2).

**Table 1:** Scores (mean ± S.D.) of the Illness Attitude Scales in healthy volunteers (controls, *n* = 129), and patients with bipolar I (BD I, *n* = 87) and II (BD II, *n* = 92) disorders

	Controls	BD I	95% Confidence Interval	BD II	95% Confidence Interval
Patho-thanatophobia	13.95 ± 8.05	12.19 ± 6.99	−3.39~1.83 vs. controls	16.86 ± 9.58a,b	1.43~6.55 vs. controls 1.96 ~ 7.58 vs. BD I
Symptom Effect	5.25 ± 3.15	7.62 ± 4.10a	1.12 ~ 3.61 vs. controls	8.36 ± 4.05a	1.91 ~ 4.35 vs. controls
Treatment Seeking	11.62 ± 3.45	13.31 ± 4.90a	.30 ~ 3.08 vs. controls	13.35 ± 4.22a	.38 ~ 3.10 vs. controls
Hypochondriacal Belief	7.23 ± 2.88	8.05 ± 3.66	−.36~1.98 vs. controls	8.74 ± 4.15a	.42 ~ 2.72 vs. controls

Note: a, *p* < .05 vs. controls; b, *p* < .05 vs. BD I

**Table 2:** Scales scores (mean ± S.D.) of personality and affective states in healthy volunteers (controls, *n* = 129), and patients with bipolar I (BD I, *n* = 86) and II (BD II, *n* = 92) disorders

	Controls	BD I	95% Confidence Interval	BD II	95% Confidence Interval
Parker Personality Measure					
Paranoid	19.07 ± 6.65	24.51 ± 5.70a	3.11~7.76 vs. controls	26.27 ± 8.27a	4.86~9.43 vs. controls
Schizoid	18.67 ± 3.78	19.68 ± 4.00	−.27~2.28 vs. controls	21.15 ± 3.76a	1.12 ~ 3.64 vs. controls
Schizotypal	8.49 ± 3.13	10.07 ± 3.13a	1.02~3.41 vs. controls	12.36 ± 4.3a,b	2.69~5.03 vs. controls .36 ~ 2.93 vs. BD I
Antisocial	17.40 ± 5.62	21.05 ± 4.99a	1.78~5.52 vs. controls	22.92 ± 6.17a	3.44~7.12 vs. controls
Borderline	18.09 ± 5.68	23.09 ± 6.60a	2.67~7.33 vs. controls	26.56 ± 8.65a,b	6.26~10.84 vs. controls 1.04 ~ 6.06 vs. BD I
Histrionic	11.47 ± 3.62	14.44 ± 3.31a	1.71~4.23 vs. controls	15.31 ± 4.40a	2.52~5.00 vs. controls
Narcissistic	14.55 ± 5.00	18.44 ± 5.11a	2.11~5.66 vs. controls	19.91 ± 5.93a	3.42~6.91 vs. controls
Avoidant	23.40 ± 7.13	27.56 ± 6.36a	1.75~6.57 vs. controls	29.41 ± 8.04a	3.74~8.48 vs. controls
Dependent	20.38 ± 5.97	24.06 ± 5.07a	1.60~5.75 vs. controls	26.34 ± 7.45a,b	4.00~8.08 vs. controls .13 ~ 4.60 vs. BD I
Obsessive-compulsive	16.34 ± 4.22	18.39 ± 3.89a	.62~3.48 vs. controls	18.00 ± 4.70a	.34~3.15 vs. controls
Passive-aggressive	18.64 ± 5.36	22.10 ± 4.95a	1.62~5.31 vs. controls	23.97 ± 6.24a	3.35~6.98 vs. controls
Plutchik-van Praag	14.84 ± 12.30	14.94 ± 11.76a	1.44 ~ 8.99 vs. controls	21.92 ± 13.58 a,b	8.51 ~ 15.88 vs. controls
Depression Inventory					2.92 ~ 11.05 vs. BD I
Mood Disorder	2.30 ± 2.56	8.33 ± 3.08a	5.11 ~ 6.94 vs. controls	5.34 ± 2.60a,b	2.14 ~ 3.93 vs. controls
Questionnaire					-3.97~-2.01 vs. BD I
Hypomanic Checklist-32	11.62 ± 5.04	19.16 ± 6.32a	5.76 ~ 9.33 vs. controls	17.66 ± 4.67a	4.29 ~ 7.79 vs. controls

Note: a, *p* < .05 vs. controls; b, *p* < .05 vs. BD I

### Group differences of affective states

The mean PVP scores were significantly different among the three groups (F [2, 306] = 31.70, *p* < .001, MSE = 3993.90, ŋ^2^ = .08). The scores in BD II were higher than those in BD I and the controls, and BD I scored higher than that in controls. Mean MDQ scores were significantly different among the three groups (F [2, 306] = 127.19, *p* < .001, MSE = 948.61, ŋ^2^ = .46); with that in BD I higher than those in BD II and the controls, and BD II scored higher than controls did. The mean HCL-32 scores were also significantly different among the three groups (F [2, 306] = 62.05, *p* < .001, MSE = 1756.77, ŋ^2^ = .29), with those in BD I and BD II higher than that in controls (also see Table 2).

### Associations between illness attitudes, personality and affective states (hypothesis 2)

When considering the prediction of IAS scales by the PERM styles and affective states, the results showed that the accounted variances (adjusted R^2^s) ranged from .05 to .20 in controls, .07 to .25 in BD I, and .26 in BD II. In controls, the Dependent style predicted Patho-thanatophobia and Symptom Effect, and Schizoid predicted Hypochondriacal Belief. In BD I, Histrionic predicted Patho-thanatophobia, HCL-32 predicted Symptom Effect, Narcissistic, Histrionic, HCL-32 and PVP together predicted Hypochondriacal Belief. In BD II, PVP and MDQ together predicted Symptom Effect, and PVP predicted Hypochondriacal Belief (Table 3).

**Table 3:** The stepwise multiple linear regression analysis for the relationships between the IAS, PERM, PVP, MDQ, and HCL-32 scales in healthy volunteers (controls, *n* = 129), and patients with bipolar I (BD I, *n* = 87) and II (BD II, *n* = 92) disorders

	a-R^2^	Controls beta (B, SE), predictor	a-R^2^	BD I beta (B, SE), predictor	a-R^2^	BD II beta (B, SE), predictor
Patho-thanatophobia	.20	.45 (.52,.09), Dependent	.07	.28 (.59,.22), Histrionic		
Symptom effect	.06	.26 (.13,.54), Dependent	.09	.30 (.19,.06), HCL-32	.26	.42 (.12,.02), PVP .26 (.41,.14), MDQ
Hypochondriacal Belief	.05	.24 (.18,.06), Schizoid	.25	−.42 (−.29,.08), Narcissistic .34 (.37,.13), Histrionic .30 (.17,.05), HCL-32 .26 (.07,.02), PVP	.26	.29 (.09,.30), PVP

Note: all predictors are significant ones at *p* < .01; a-R^2^, adjusted R^2^; B, beta; SE, Standardized error

## Discussion

The current study was designed to look for the differences of hypochondriasis and their associations with affective states and personality variables in two types of bipolar disorder, and our results might help to explain the treatment-difficulty of the disorder. Compared to healthy controls, BD I and BD II scored significantly higher on MDQ, HCL-32, and PVP scales, on almost all PERM styles, and on IAS Symptom Effect and Treatment Seeking; and their score differences on PVP and IAS were relatively weak as indicated by the effect sizes (ŋ^2^: .08). Specifically, BD II scored higher on Patho-thanatophobia and Hypochondriacal Belief than healthy controls, and also scored higher on Patho-thanatophobia than BD I. The results confirm previous reports that hypochondriac features are prominent in bipolar disorders [20], and confirmed our first hypothesis. Regarding associations with IAS scales, affective states were involved in both BD I and BD II, but some disordered personality symptoms or traits were in addition involved in BD I, which confirmed our second hypothesis. Our results therefore demonstrate that the belief regarding health is associated with personality symptoms, and that it mutually influenced the affective states, which supported the view that the implicit theories play a role in the etiopathology of bipolar disorder.

The higher scores of MDQ, HCL-32 and PVP in BD I and BD II were consistent with previous studies [24, 25]. Moreover, both BD I and BD II exhibited more comorbid personality disorders as they scored higher on most PERM scales, consistent with the previous reports [24, 35, 36]. BD II also scored higher on Borderline, Schizotypal and Dependent scales than BD I did in our study. Considering that patients with these personality disorders display more distressed emotions [23], our results are consistent with the description that BD II is associated with a high level of neuroticism [37] and explain its treatment difficulty due to the involvement of the pathological personality styles.

Previous results show that in manic or hypomanic states, hypochondriasis can be a manifestation of delusions or distorted beliefs in bipolar disorder [19]. The documentation in general explains the higher IAS scale scores in our BD I and BD II. The higher IAS scale scores in BD I and BD II were also consistent with a previous report that both patients with hypochondriasis and bipolar disorder showed cognitive dysfunctions [38]. For example, in bipolar disorder patients, individuals’ defenses tend to indicate an increase in hypochondriasis when manic episodes begin to abate [21], while in depressive patients, individuals reported difficulties in describing feelings that specifically predict the degree of health anxiety and illness-related behaviors [39]. All these help to explain the higher score of IAS in bipolar disorder.

Patho-thanatophobia is the core characteristic of hypochondriasis, which covers the fears of death and dying [40] and is closely associated with anxiety [41]. BD II patients exhibit more lifetime phobia and other anxiety disorders [4, 42], which might account for the higher Patho-thanatophobia in BD II than that in BD I. Moreover, BD II scored higher on Dependent, Schizotypal and Borderline styles than BD I did, which supports the higher suspiciousness [43] and anxiety [8, 23] in BD II, and helps to explain the higher Patho-thanatophobia and Hypochondriacal Belief in BD II. Therefore, both our study and the literature show that the influences between illness attitudes and the affective states in bipolar disorder are reciprocal, which fits into the implicit theories.

In controls, the Dependent style predicted Patho-thanatophobia and Symptom Effect, which is consistent with the prevalence of dependent personality disorder in hypochondriac patients [23]. Other studies have shown that patients with dependent personality disorder are anxious [44], report more clinical symptoms, and rely more on doctors or medication in life or in the hospital [45], and thus are more apt to seek treatment, and use health services than patients without personality disorders [46]. There are also some related studies supporting that the Schizoid style predicts Hypochondriac Belief. Both schizoid and avoidant factors resided in the schizophrenia spectrum disorders [47], which have overlapping clinical features with each other [9], and avoidant personality disorder is often found in hypochondriasis [22, 23]. These observations indirectly point to the association between Schizoid and Hypochondriacal Belief.

In BD I, Narcissistic was negatively associated with Hypochondriacal Belief which was consistent with the clinical manifestations of narcissistic personality disorder. For instance, narcissistic patients are characterized by attention seeking or self-esteem demanding, in order to reduce their psychosocial distress, and then to reduce clinical hypochondriasis [23, 48, 49]. The mania helps these patients to elevate the self-esteem, thus present less distress and less hypochondriasis. On the other hand, the association between Histrionic and Patho-thanatophobia and Hypochondriacal Belief was consistent with the clinical features of histrionic personality patients, for instance the high rate of somatic disorders, conversion disorder, major depressive disorder [9], and increased anxiety disorder comorbidity [50], all of which are closely related to hypochondriasis. Previous results also pointed to the greater hypochondriac concerns in histrionic personality disorder [51]. Our results suggest that the pathological personality styles, such as Narcissistic and Histrionic which held different cognitions or beliefs, display different associations with the health-related anxiety or hypochondriasis in BD I.

In BD II, affective states have great effect on hypochondriasis. Regarding the association between PVP and Symptom Effect and Hypochondriacal Belief, previous studies showed that depression in BD II is more severe than that in BD I, especially somatic symptoms [5], which were linked to Symptom Effect, with BD II associated with increased retardation, guilt delusions, and suicidal ideations [3] which are linked with increased hypochondriacal belief. Generally, bipolar disorder patients mobilize their ego defense through mania to decrease their health-related symptoms, but they are still symptomatic and hypochondriac [21]. This paradox might be responsible for the association between MDQ and Symptom Effect found in our BD II.

There are four limitations of the present study. Firstly, our participants were between 17 and 49 years of age and so the current results might not be generalizable for elderly individuals, since hypochondriasis is ubiquitous in this population [52]. Secondly, our study lacked other disease controls such as unipolar depression, schizophrenia, personality disorder, or health-related anxiety disorder. Thirdly, we used self-report questionnaires, which might result in measurement profile-floating. Fourthly, our study was a cross-sectional design, which lacks a longitudinal perspective and precludes a conclusion about the causal-effective nature between these measures in a given group. Further comparisons with more clinical controls or conducting a cohort design would clear up our current associations.

## Conclusions

Our study indicates the presence of more hypochondriac symptoms in bipolar disorder, especially BD II, and that the increased hypochondriasis has contributions from disordered personality symptoms. Specifically, mania in BD I and depression in BD II are predictive of some hypochondriac aspects, and personality symptoms contribute to the association between mood and hypochondriasis in BD I and more so in healthy controls. Our results thus offer more explanations for the treatment-recalcitrant features of bipolar disorder, especially of BD II.

## Abbreviations

BD I: Bipolar I disorder
BD II: Bipolar II disorder
CT: Computed tomography
MRI: Magnetic resonance imaging
IAS: The Illness Attitude Scale
PERM: The Parker Personality Measure
MDQ: The Mood Disorder Questionnaire
HCL-32: The Hypomania Checklist-32
PVP: The Plutchik - van praag Depression Inventory
